# Interleukin-18 as a Potential Biomarker for Radiotherapy-Related Pain in Breast Cancer: Implications for Personalized Pain Management

**DOI:** 10.3390/cancers18071100

**Published:** 2026-03-28

**Authors:** Alexandra N. McMahon, Juan Pablo de Rivero Vaccari, Isildinha M. Reis, Cristiane Takita, Jean L. Wright, Yan Guo, Jennifer J. Hu

**Affiliations:** 1 Department of Public Health Sciences, University of Miami Miller School of Medicine, Miami, FL 33136, USA; axm8143@miami.edu (A.N.M.);; 2 Department of Neurological Surgery and The Miami Project to Cure Paralysis, University of Miami Miller School of Medicine, Miami, FL 33136, USA; jderivero@med.miami.edu; 3 Sylvester Comprehensive Cancer Center, University of Miami Miller School of Medicine, Miami, FL 33136, USA; 4 Department of Radiation Oncology, University of Miami Miller School of Medicine, Miami, FL 33136, USA; 5 Department of Radiation Oncology, University of North Carolina School of Medicine, Chapel Hill, NC 27514, USA

**Keywords:** IL-18, inflammasome, breast cancer, radiotherapy, pain

## Abstract

**Background:** Adjuvant RT remains a standard therapy for breast cancer, reducing recurrence risk and improving survival; however, it can also induce side effects, including pain. Inflammasome-related biomarkers, such as interleukin-18 (IL-18), play a role in inflammation-mediated pain, and we hypothesize that IL-18 may serve as a potential biomarker for breast cancer RT-induced pain. **Methods:** The association between IL-18 and pain was assessed among breast cancer patients receiving adjuvant RT. Plasma IL-18 protein concentration was quantified before and after RT using Ella SimplePlex technology (Biotechne). Clinically relevant pain outcomes included pre-RT pain (pain score ≥ 4), post-RT pain (pain score ≥ 4), and RT-related pain (increase in pain from <4 pre-RT to ≥4 post-RT). Multivariable logistic regression assessed the association between IL-18 and pain outcomes, adjusting for demographic and treatment-related factors. The joint effect of IL-18 and obesity on pain were also explored. **Results:** Patients in the highest pre-RT IL-18 quartile experienced higher odds of both post-RT pain (OR = 2.36, 95% CI: 1.15–4.87) and RT-related pain (OR = 2.73, 95% CI: 1.20–6.26). IL-18 levels increased from pre-RT to post-RT with a mean change of 0.07 (SD = 0.35). In addition, patients with elevated pre-RT IL-18 levels and obesity experienced the highest odds of post-RT pain (OR = 3.97, 95% CI: 1.98–7.98) and RT-related pain (OR = 2.84, 95% CI: 1.32–6.09), suggesting a potential combined effect. **Conclusions:** Elevated pre-RT IL-18 levels were associated with an increased risk of pain following adjuvant RT, particularly in breast cancer patients with obesity. Thus, IL-18 may serve as a potential biomarker for identifying patients at increased risk for RT-related pain and informing treatment decision.

## Simple Summary

Interleukin (IL)-18 is a pro-inflammatory cytokine involved in inflammation and immune activation and plays a role in treatment-related pain. The present study investigates the association between IL-18 and pain in a large prospective cohort of breast cancer patients receiving adjuvant radiotherapy (RT). IL-18 levels and perceived pain were measured before and after RT. Study findings indicate that patients with higher IL-18 levels before treatment were more likely to experience RT-related pain. Moreover, the risk was even greater among patients with obesity, suggesting that inflammation and metabolic factors may jointly increase pain during RT. These results suggest that IL-18 may be a useful biomarker for identifying breast cancer patients at higher risk for RT-related pain and for supporting potential precision-intervention strategies targeting inflammation.

## 1. Introduction

Breast cancer remains a significant public health priority as one of the most frequently diagnosed cancers and a leading cause of cancer-related mortality in women. In 2026, an estimated 324,580 new cases and 42,670 breast cancer-related deaths are projected [1]. Advances in screening and treatment strategies for breast cancer including systemic therapies, and RT, have improved patient prognosis and survival [2]. However, many of these advances involve the use of adjuvant therapies that improve disease control and overall survival but may also produce treatment-related side effects. Among these therapies, RT is widely used to reduce local reoccurrence and improve clinical outcomes.

Despite clinical benefits, RT can lead to both acute and chronic toxicities, including skin reactions, fibrosis, lymphedema, fatigue, and pain [3]. RT-related pain is a particularly burdensome side effect for those who experience it, as it may persist after treatment completion, thus impacting patients’ quality of life (QOL). The severity and presentation of RT-related symptoms vary across individuals, suggesting that underlying clinical and biological factors may influence treatment response. Importantly, treatment-related pain experienced during RT does not affect all patients equally, and some evidence suggests biological mechanisms, including inflammatory and immune responses may contribute to pain sensitivity in cancer populations [4,5].

There is growing interest in the role of chronic inflammation in cancer treatment responses and symptom burden, including treatment-related pain. Inflammation is produced by innate immune mechanisms such as inflammasomes [6], which are multiprotein complexes that regulate the activation of pro-inflammatory cytokines, including interleukin (IL)-18 and IL-1β [7]. IL-18 is a cytokine involved in inflammatory responses, immune signaling and tissue injury [8]. RT induces cellular damage activating repair mechanisms and inflammasome pathways, leading to release of pro-inflammatory cytokines IL-18 [9,10]. These processes may promote pain and nociceptive sensitization, and the proposed pathway is illustrated in Supplementary Figure S1.

Various studies have shown associations between IL-18 and pain, including cancer-related, chronic diseases and neuropathic pain [11,12,13]. Furthermore, there is growing evidence to suggest that IL-18 is upregulated in chronic pain, thus inhibiting IL-18 has the potential to alleviate pain [11]. However, there is limited evidence exploring the role of inflammasome activation in pain and RT-related side effects among breast cancer patients. Our lab has previously reported an association between C-reactive protein (CRP) and RT-related pain in breast cancer [14]; however, IL-18 has been primarily studied in systemic inflammation and cancer progression research rather than RT-related pain outcomes.

Clinical and metabolic factors may further promote inflammasome activation, influencing treatment efficacy and treatment-related toxicities in breast cancer. Obesity is of particular interest, as it is associated with both systemic inflammation and increased treatment-related toxicities and symptom burden in breast cancer [15,16]. It has been shown that adipose tissue releases proinflammatory mediators, activating the inflammasome and downstream cytokine signaling including IL-6 and tumor necrosis factor (TNF) [17], potentially amplifying pain during cancer treatment. Thus, it is critical to better understand the combined metabolic and inflammatory factors underlying treatment related pain.

Therefore, the present study aims to address these gaps by exploring the association between IL-18 and pain in breast cancer patients undergoing adjuvant RT. In addition, we explore the combined effects of inflammatory and metabolic factors on RT-related pain. Importantly, by identifying biomarkers associated with elevated pain risk, our findings may help guide treatment decisions and precision medicine strategies to improve pain during RT.

## 2. Materials and Methods

### 2.1. Study Design and Patient Population

Clinical data and plasma samples for the current analysis are from a prospective cohort study at the University of Miami, FL, USA, as detailed previously [14,18]. In brief, patients were recruited at the University of Miami Sylvester Comprehensive Cancer Center and Jackson Memorial Hospital in Miami, Florida, between December 2008 and August 2014. Institutional Review Board approval was received, and patients completed written informed consent. Study participants were female adults (≥18 years at diagnosis) newly diagnosed with AJCC stage 0–III breast cancer who received breast conserving surgery and planned to receive adjuvant RT. All study participants were able to speak English or Spanish. Patients receiving concurrent chemoradiation or partial breast irradiation were excluded from the study. RT was delivered following prevailing practices at the time of study including the use of standard or partially wide photon tangents with 6 and/or 10 MV photons. RT was administered through conventional fractionation (total dose 45–50 Gy, dose per fraction 1.8–2.0 Gy) or hypo-fractionation (2.66–2.67 Gy/day over 3 weeks) to the whole breast with or without regional lymph nodes with some patients receiving an additional boost dose of 10–20 Gy without bolus. Radiation oncologists contoured target volumes, including the breast and lumpectomy cavity. Participants with missing IL-18 or pain measurements at pre- or post-RT were excluded from the corresponding regression analyses. Among the 372 participants with pre- and post-RT IL-18 measurements, 333 patients had both pre- and post-RT pain assessments.

### 2.2. Assessment of Pain

All patients enrolled in the parent study completed the National Surgical Adjuvant Breast and Bowel Project (NSABP) B-39/RTOG 0413 protocol QOL questionnaire at pre- and post-RT visits. This questionnaire measured treatment-related symptoms, breast cosmesis, fatigue, and perceived convenience of care. The section assessing treatment-related symptoms included four pain severity items from the Brief Pain Inventory (BPI): “Rate your pain at its worst, at its least, on average in the past four weeks, and now (0 = no pain to 10 = pain as bad as you can imagine)”. The present study used an average of these four items to classify pre- and post-RT pain, where a score of 4–10 was defined as clinically relevant pain based on prior studies [14,19]. RT-related pain was defined as an increase in pain score from <4 pre-RT to ≥4 from post-RT. Pain scores were captured from a questionnaire administered on the day before RT initiation and on the day after RT completion.

### 2.3. Assessment of IL-18

Blood samples were collected at pre- and post-RT and were processed within 2 hours for isolation of plasma. The aliquoted plasma samples were then stored at − 80 °C until assay. Plasma IL-18 levels were measured using the Ella Simple Plex™ immunoassay platform (ProteinSimple, San Jose, CA, USA) as described in [20]. This methodology has been validated in past studies [21,22]. In brief, samples were analyzed at a 2-fold dilution, and mean fluorescence intensity values were converted to concentrations using a five-parameter logistic (5-PL) standard curve for each assay. All assays were performed using cartridges from the manufacturer’s specifications, and data acquisition and analysis were carried out using Ella Runner software (version 4.1.0.22; Bio-Techne, Minneapolis, MN, USA) as in [23]. The Ella Simple Plex assay has a lower limit of quantification of approximately 0.96 pg/mL for IL-18 on the original concentration scale. To ensure accuracy, intra-assay variability was assessed by the percent coefficient of variation (%CV) across replicate measurements where most samples showed acceptable variation. Samples with concentrations outside the assay detection range were rerun. Samples were analyzed in batches based on collection timing and paired pre- and post-radiotherapy samples were processed within the same batch when possible. Potential batch effects were evaluated by comparing mean IL-18 levels across assay batches using analysis of variance (ANOVA).

### 2.4. Statistical Analysis

Descriptive statistics summarized the demographic, clinical, and treatment characteristics of the study population. IL-18 concentrations were log-transformed before analyses to improve normality. First, chi-square tests or Fisher’s exact tests were used to compare clinical and demographic variables by pain status (pre-RT pain, post-RT pain, and RT-related pain). One-way ANOVA or independent samples t-tests were used to evaluate differences in IL-18 levels by demographic and clinical characteristics. IL-18 levels within categories of covariates were summarized using mean and standard deviation (SD) at pre-RT, post-RT, and change by RT.

For regression analyses, the effect of IL-18 was assessed using both continuous (incorporating linear and quadratic terms to test for non-linearity) and categorical (quartiles) specifications based on its distribution for pre-RT, post-RT and change by RT. Multivariable logistic regression was used to assess the relationship between IL-18 and the following binary pain outcomes: pre-RT pain (pain score ≥ 4 before RT), post-RT pain (pain score ≥ 4 after RT), and RT-related pain (increase in pain from <4 pre-RT to ≥4 post-RT). Covariates included in multivariable models were selected based on prior literature, clinical relevance, and statistical evaluation. The final adjusted multivariable model included age at diagnosis, race/ethnicity, clinical tumor stage, RT fractionation, and either or both BMI and IL-18. To evaluate the potential combined effects of obesity and IL-18 on pain outcomes, joint exposure categories were created for BMI (<30 vs. ≥30 kg/m^2^) and binary IL-18 (dichotomized at the median). Interaction between BMI and IL-18 was also evaluated using a multiplicative interaction term (BMI × IL-18) in multivariable logistic regression models.

## 3. Results

### 3.1. Pain Outcomes by Demographic and Clinical Characteristics

Table 1 presents the distribution of pain outcomes by clinical and demographic characteristics. Seventeen percent (*n* = 60) of patients experienced pain before RT, 32% (*n* = 112) reported pain following RT, and 23% (*n* = 78) experienced RT-related pain. Pre-RT pain differed by race/ethnicity (*p* = 0.028) and HER2 status (*p* = 0.032). While post-RT pain was more common among younger patients (<50 years vs. ≥50 years: 45% vs. 27%; *p* = 0.001), obese patients (42% vs. 30% overweight and 21% normal weight; *p* = 0.004), patients with stage III disease (46% vs. 29% for stage 0–II; *p* = 0.009), those receiving ≥60 Gy (38% vs. 23%; *p* = 0.012), and those treated with conventional fractionation compared to hypofractionation (34% vs. 19%; *p* = 0.046). Whereas for RT-related pain, patients younger than 50 years (33% vs. 19%; *p* = 0.006), patients with more advanced-stage disease (35% vs. 21%; *p* = 0.012), and those receiving conventional fractionation (25% vs. 11%; *p* = 0.042) were more likely to experience increase in pain during treatment.

### 3.2. IL-18 Levels by Demographic and Clinical Characteristics

Table 2 shows the distribution of pre-RT, post-RT and change by RT IL-18 levels (Log10-transformed) by clinical and demographic characteristics. Mean IL-18 levels increased from pre-RT to post-RT with a change of 0.07 (SD = 0.35). For pre-RT IL-18 levels, there were significant differences by race/ethnicity and clinical tumor stage (*p* = 0.035 and *p* = 0.038 respectively). For post-RT IL-18 levels, higher levels were observed among HER2-positive patients (mean = 5.41 vs. 5.25 for HER2-negative; *p* = 0.023), patients receiving ≥60 Gy (mean = 5.32 vs. 5.19; *p* = 0.015), and those treated with conventional fractionation (mean = 5.30 vs. 5.12; *p* = 0.013). Additionally, the change in IL-18 from pre- to post-RT was greater among patients receiving ≥60 Gy (mean change = 0.09 vs. 0.00; *p* = 0.032).

### 3.3. Association Between IL-18 Levels and Pain Outcomes

Table 3 shows the relationship between IL-18 and pain outcomes in the multivariable adjusted models. Patients in the two highest pre-RT IL-18 quartiles presented increased odds of post-RT pain after adjustments (OR = 3.35, 95% CI: 1.64–6.85 and OR = 2.36, 95% CI: 1.15–4.87, respectively). Similarly, patients in the two highest pre-RT IL-18 quartile had increased odds of RT-related pain compared to the reference first quartile (OR = 3.22, 95% CI: 1.44–7.20 and OR = 2.73, 95% CI: 1.20–6.26, respectively), further highlighting the potential association between pre-RT IL-18 and treatment-related pain.

Receiver operating characteristic (ROC) analyses were conducted to further test the discriminatory ability of IL-18 in pain outcomes (Supplementary Figures S2–S4). For post-RT pain, the AUC increased from 0.679 for the clinical model alone to 0.733 for the model including clinical covariates, body mass index, and IL-18 quartiles. Similar improvements were observed for pre-RT pain and RT-related pain outcomes, with the addition of IL-18 resulting in modest increases in AUC values.

### 3.4. Combined Effects of IL-18 and Obesity on Pain Outcomes

Given the observed associations between obesity, IL-18 levels, and pain outcomes, we further examined whether the combined effects of obesity and elevated IL-18 were associated with higher odds of pain than either variable independently (Table 4). Obesity was independently associated with increased odds of pre-RT pain (OR = 2.04, 95% CI: 1.14–3.64) and post-RT pain (OR = 2.20, 95% CI: 1.34–3.61). Elevated pre-RT IL-18 levels (≥5.20 pg/mL) were associated with increased odds of post-RT pain (OR = 2.03, 95% CI: 1.24–3.32) and RT-related pain (OR = 2.25, 95% CI: 1.29–3.93). From the combined analysis, obese patients with elevated pre-RT IL-18 had the highest odds of pain outcomes, including pre-RT pain (OR = 2.22, 95% CI: 1.03–4.78), post-RT pain (OR = 3.97, 95% CI: 1.98–7.98), and RT-related pain (OR = 2.84, 95% CI: 1.32–6.09). Similarly, patients with obesity and higher post-RT IL-18 levels experienced greater odds of both pre- and post-RT pain. In validation analyses, multiplicative interaction terms between obesity and IL-18 were not statistically significant for pain outcomes. Thus, findings presented in Table 4 reflect combined exposure groups rather than multiplicative statistical interaction.

## 4. Discussion

Adjuvant RT is frequently utilized in breast cancer care as it plays a critical role in reducing recurrence and improving survival. Despite clinical benefits, RT can induce various side effects that negatively impact the QOL of patients and treatment adherence [24]. Thus, it is critical to explore possible predictors of more severe side effects in response to RT. In the present study of breast cancer patients receiving adjuvant RT, we explored the association between inflammatory marker IL-18 and RT-related pain. To the best of our knowledge, this is the first study to evaluate IL-18 in breast cancer RT-related pain.

The findings from the current study demonstrated an association between higher pre-RT IL-18 levels and RT-related pain in breast cancer. IL-18 is a proinflammatory cytokine produced from inflammasome activation and plays a critical role in immune regulation and tissue injury [8]. Prior studies have shown that IL-18 plays a role in modulating peripheral and central inflammatory pathways, which may influence pain signaling [11]. As pre-RT IL-18 levels were associated with post-RT pain, study findings suggest that pre-existing inflammation may be an important predictor of RT-related pain. This is aligned with current clinical understanding since patients with elevated IL-18 at baseline may have sensitized nociceptive pathways, potentially making them more vulnerable to RT-related pain. Additionally, IL-18 levels may also reflect broader inflammasome or immune dysregulation [25], which may predispose patients to pain potentially independent of RT-induced cytokine changes.

Breast cancer patients with obesity and elevated IL-18 levels experienced the highest odds of pre-RT pain, post-RT pain, and RT-related pain. The strong association observed between patients with high BMI and IL-18 levels suggests that metabolic dysregulation and inflammatory activity may have a dual effect on pain. This combined effect may increase inflammasome activation and pain. These findings are also supported by prior studies that have demonstrated increased treatment-related toxicities among breast cancer patients with obesity [26,27,28], providing further evidence of the potential effects of obesity-related inflammation and IL-18 on RT-related pain.

The present study offers important clinical insight. First, the association observed between pre-RT IL-18 levels and RT-related pain encourages future research on the implementation of targeted pain management protocols—such as prophylactic anti-inflammatory therapy or intensive monitoring—prior to the start of RT. Some studies have reported that reductions in IL-18 expression may alleviate pain [29], which is supported by our study findings. Further evidence suggests that targeting inflammasome pathways, including IL-18 and NLRP3, through strategies including pharmacologic inhibitors [30] and topical protective agents may help reduce radiation-induced tissue injury and inflammation [31,32,33]. Therefore, our findings encourage additional exploration of potential interventions targeting inflammation and IL-18 reduction in pain management. Further, the combined effects of IL-18 and obesity highlight the importance of considering the joint effects of metabolic and inflammatory factors when assessing treatment-related symptom risk. However, findings from the present study are exploratory and future research is needed in larger, longitudinal cohorts to better understand the relationship between IL-18 and breast cancer-related pain and to define clinically meaningful changes in IL-18 levels. As the field of RT progresses, pre-RT biomarker-informed pain (e.g., IL-18) may help dose adaptation to maximize tumor control while minimizing toxicity.

There are various strengths and limitations to the current study. To begin, the study captures biological samples and clinical data from a highly diverse prospective breast cancer cohort. Further, to minimize potential recall bias, biological samples and patient-reported QOL outcomes were both collected on the first and last day of RT. However, it is important to note that moderate hypofractionation is the standard of care for most patients, and pain is typically reduced with this approach. Additionally, pain assessed in this analysis reflects acute pain at the completion of RT rather than long-term chronic pain. Variables including the usage of pain medication or anti-inflammatory agents were not available and would be useful to explore in future research. This study was also conducted at a single center, which may limit generalizability of the findings. In future studies, it may be beneficial to perform additional repeated measures to better capture changes in health status and longitudinal trends in IL-18 and pain. Lastly, exploring whether pain is nociceptive or neuropathic in future research could provide further insight into how varying pain types may be influenced by inflammatory activity.

## 5. Conclusions

In this prospective cohort of breast cancer patients receiving adjuvant RT, IL-18 was associated with RT-related pain, particularly among obese patients, suggesting that inflammasome-mediated pathways may contribute to RT-related pain, consistent with previous studies showing the role of the inflammasome in pain [34]. Study findings highlight the importance of inflammatory and metabolic factors in RT-related breast cancer symptom burden and may support the development of personalized pain management strategies including targeting IL-18 to improve QOL and pain burden in breast cancer prior to the start of RT.

## Figures and Tables

**Table 1:** Clinically Significant Pain by Demographic and Clinical Characteristics.

Variables	Pre-RT Pain (≥4)					Post-RT Pain (≥4)					RT-Related Pain ^1^ (Change from <4 to ≥4)				
	No		Yes			No		Yes			No		Yes		
	*n*	%	*n*	%	*p*	*n*	%	*n*	%	*p*	*n*	%	*n*	%	*p*
**Total**	284	83	60	17		236	68	112	32		255	77	78	23	
**Age at Diagnosis**					0.748					**0.001**					**0.006**
<50	84	82	19	18		58	55	47	45		69	67	34	33	
≥50	200	83	41	17		178	73	65	27		186	81	44	19	
Mean (SD)	54.8 (9.4)		53.5 (9.5)			55.7 (9.1)		52.2 (9.8)			55.3 (9.1)		51.7 (9.9)		
**Race/Ethnicity**					**0.028**					**0.007**					0.087
NHW	46	98	1	2		41	85	7	15		40	89	5	11	
HW	178	80	44	20		145	64	80	36		160	74	55	26	
AA	51	81	12	19		39	62	24	38		44	72	17	28	
Other	9	75	3	25		11	92	1	8		11	92	1	8	
**BMI**					0.055					**0.004**					0.055
Normal	74	86	12	14		70	79	19	21		74	86	12	14	
Overweight	88	73	32	27		90	70	38	30		88	73	32	27	
Obese	93	73	34	27		76	58	55	42		93	73	34	27	
**Clinical Tumor Stage**					0.369					**0.009**					**0.012**
0-II	232	83	46	17		202	71	83	29		215	79	56	21	
III	52	79	14	21		34	54	29	46		40	65	22	35	
**ER**					0.148					0.961					0.231
Negative	72	88	10	12		53	68	25	32		55	71	22	29	
Positive	211	81	50	19		182	68	87	32		199	78	56	22	
**PR**					0.604					0.542					0.231
Negative	104	84	20	16		83	70	36	30		86	74	31	27	
Positive	178	82	40	18		151	67	76	33		167	78	47	22	
**HER2**					**0.032**					0.054					0.053
Negative	211	83	43	17		180	68	83	32		194	78	56	22	
Positive	29	69	13	31		20	53	18	47		24	63	14	37	
**Triple negative**					0.101					0.964					0.346
No	224	80	55	20		191	67	94	33		209	77	62	23	
Yes	45	90	5	10		33	67	16	33		34	71	14	29	
**Chemotherapy**					0.429					0.064					0.054
No	113	84	21	16		103	73	38	27		108	82	24	18	
Yes	166	81	39	19		129	64	74	36		143	73	54	27	
**Total RT dose (Gy)**					0.125					**0.012**					0.092
<60	70	88	10	13		64	77	19	23		64	82	14	18	
≥60	186	80	47	20		145	62	89	38		163	72	62	28	
**RT Fractionation**					0.396					**0.046**					**0.042**
Conventional	245	82	55	18		198	66	104	34		217	75	74	25	
Moderate Hypo	34	87	5	13		34	81	8	19		34	89	4	11	

Note. *p* values from the chi-square test or Fisher’s Exact Test. NOTE. Boldface indicates significance at *p* < 0.05. IL-18 values were log-transformed prior to analysis to ensure normality. ^1^ Patients who reported an increase in pain level from pre- to post-RT (i.e., pain score changed from <4 to ≥4) were defined as having RT-related pain. Abbreviations: AA, African American; HW, Hispanic White; NHW, Non-Hispanic White; BMI, body mass index; HER2, human epidermal growth factor receptor 2; RT, radiotherapy; SD, standard deviation; Hypo = hypofractionation.

**Table 2:** IL-18 Levels by Demographic and Clinical Characteristics.

Variables	Pre-RT IL-18				Post-RT IL-18				Change by RT IL-18			
	*n*	Mean	SD	*p*	*n*	Mean	SD	*p*	*n*	Mean	SD	*p*
**Total**	372	5.20	0.46		372	5.27	0.44		372	0.07	0.35	
**Age at Diagnosis**				0.960				0.683				0.560
<50	110	5.20	0.39		110	5.29	0.41		110	0.08	0.40	
≥50	262	5.20	0.49		262	5.27	0.46		262	0.06	0.33	
**Race**				**0.035**				**0.014**				0.088
NHW	51	5.23	0.45		51	5.22	0.48		51	0.00	0.35	
HW	241	5.21	0.45		241	5.31	0.42		241	0.10	0.35	
AA	68	5.13	0.49		68	5.14	0.42		68	0.01	0.34	
Other	12	5.38	0.70		12	5.42	0.69		12	0.04	0.41	
**BMI**				0.949				0.057				0.127
Normal	96	5.19	0.47		96	5.19	0.45		96	0.01	0.34	
Overweight	136	5.19	0.48		136	5.27	0.43		136	0.08	0.36	
Obese	140	5.23	0.45		140	5.32	0.45		140	0.10	0.33	
**Clinical Tumor Stage**				**0.038**				0.495				0.059
0-II	302	5.23	0.48		302	5.28	0.45		302	0.05	0.35	
III	70	5.10	0.39		70	5.24	0.43		70	0.14	0.34	
**ER**				0.228				0.815				0.056
Negative	88	5.26	0.49		88	5.26	0.48		88	0.01	0.32	
Positive	283	5.19	0.46		283	5.28	0.44		283	0.09	0.35	
**PR**				0.301				0.672				0.403
Negative	132	5.23	0.49		132	5.29	0.49		132	0.05	0.34	
Positive	238	5.18	0.45		238	5.26	0.42		238	0.08	0.35	
**HER2**				0.402				**0.023**				0.078
Negative	276	5.20	0.45		276	5.25	0.43		276	0.05	0.35	
Positive	46	5.26	0.44		46	5.41	0.51		46	0.15	0.41	
**Triple negative**				0.610				0.509				0.136
No	305	5.20	0.45		305	5.28	0.43		305	0.08	0.35	
Yes	52	5.23	0.52		52	5.24	0.51		52	0.00	0.37	
**Chemotherapy**				0.149				0.683				0.152
No	152	5.16	0.50		152	5.26	0.44		152	0.10	0.36	
Yes	220	5.23	0.44		220	5.28	0.44		220	0.05	0.34	
**Total RT Dose (Gy)**				0.550				**0.015**				**0.032**
<60	88	5.19	0.55		88	5.19	0.45		88	0.00	0.32	
≥60	251	5.23	0.43		251	5.32	0.42		251	0.09	0.35	
**RT Fractionation**				**0.035**				**0.013**				0.713
Conventional	324	5.22	0.45		324	5.30	0.44		324	0.07	0.35	
Moderate Hypo	43	5.06	0.54		43	5.12	0.49		43	0.05	0.34	

Note. *p* values from *t*-tests and ANOVA; Boldface indicates significance at *p* < 0.05. IL-18 values were log10-transformed prior to analysis to ensure normality. Abbreviations: AA, African American; HW, Hispanic White; NHW, Non-Hispanic White; BMI, body mass index; HER2, human epidermal growth factor receptor 2; RT, radiotherapy; SD, standard deviation; Hypo = hypofractionation.

**Table 3:** Association of IL-18 Quartiles with Pre-RT, Post-RT, and RT-Related Pain.

	Pre-RT Pain (≥4) (Events = 60/*n* = 344)		Post-RT Pain (≥4) (Events = 112/*n* = 348)		RT-Related Pain (Change from <4 to ≥4) (Events = 78/*n* = 333)	
IL-18, in Quartiles	OR (95% CI)	*p*	OR (95% CI)	*p*	OR (95% CI)	*p*
**Pre-RT**						
3.94–4.93	**Referent**		**Referent**		**Referent**	
4.93–5.20	2.25 (0.95, 5.35)	0.067	1.75 (0.86–3.59)	0.125	1.71 (0.74–3.96)	0.208
5.20–5.48	**2.37 (1.02, 5.50)**	**0.045**	**3.35 (1.64, 6.85)**	**0.001**	**3.22 (1.44, 7.20)**	**0.005**
5.48–6.95	1.47 (0.59, 3.62)	0.407	**2.36 (1.15–4.87)**	**0.019**	**2.73 (1.20, 6.26)**	**0.017**
**Post-RT**						
3.95–4.99	**Referent**		**Referent**		**Referent**	
5.00–5.25	2.00 (0.79, 5.09)	0.145	0.90 (0.45, 1.83)	0.779	0.93 (0.42, 2.09)	0.864
5.26–5.51	**2.48 (1.00, 6.17)**	**0.050**	1.77 (0.89, 3.49)	0.102	1.59 (0.74, 3.41)	0.235
5.51–7.33	2.06 (0.82, 5.20)	0.126	1.45 (0.73, 2.88)	0.293	1.54 (0.71, 3.33)	0.273
**Change by RT**						
−1.17–0.14	**Referent**		**Referent**		**Referent**	
−0.14–0.05	1.29 (0.57, 2.94)	0.540	1.45 (0.73, 2.86)	0.286	1.20 (0.55, 2.62)	0.647
0.05–0.29	1.05 (0.46, 2.41)	0.902	1.14 (0.57, 2.26)	0.714	1.52 (0.71, 3.26)	0.288
0.29–1.65	1.05 (0.46, 2.41)	0.906	1.02 (0.51, 2.05)	0.953	1.08 (0.49, 2.39)	0.848

Note. Pre-RT pain was defined as a pre-RT pain score ≥ 4, post-RT pain as a post-RT pain score ≥ 4, and RT-related pain as an increase from <4 pre-RT to ≥4 post-RT. Odds ratios (ORs) and 95% confidence intervals (CIs) were obtained from multivariable logistic regression models adjusted for race/ethnicity, age group, RT fractionation and clinical tumor stage. IL-18 values were log10-transformed and categorized into quartiles. Boldface indicates significance at *p* < 0.05.

**Table 4:** Combined Effects of IL-18 and Obesity on Pre-RT, Post-RT, and RT-Related Pain.

		Pre-RT Pain (≥4) (Events = 60/*n* = 344)		Post-RT Pain (≥4) (Events = 112/*n* = 348)		RT-Related Pain (Change from <4 to ≥4) (Events = 78/*n* = 333)	
**BMI, kg/m^2^**	**Pre-RT IL-18, pg/mL**	**OR (95% CI)**	***p***	**OR (95% CI)**	***p***	**OR (95% CI)**	***p***
<30	NA	Referent		Referent		Referent	
≥30	NA	**2.04 (1.14, 3.64)**	**0.016**	**2.20 (1.34, 3.61)**	**0.002**	1.39 (0.80, 2.39)	0.242
NA	<5.20	Referent		Referent		Referent	
NA	≥5.20	1.19 (0.67, 2.11)	0.563	**2.03 (1.24, 3.32)**	**0.005**	**2.25 (1.29, 3.93)**	**0.004**
<30	<5.20	Referent		Referent		Referent	
<30	≥5.20	0.88 (0.39, 2.00)	0.756	**2.17 (1.12, 4.19)**	**0.021**	**2.44 (1.19, 5.01)**	**0.015**
≥30	<5.20	1.58 (0.68, 3.67)	0.293	**2.43 (1.17, 5.04)**	**0.017**	1.49 (0.64, 3.48)	0.354
≥30	≥5.20	**2.22 (1.03, 4.78)**	**0.041**	**3.97 (1.98, 7.98)**	**<.001**	**2.84 (1.32, 6.09)**	**0.007**
**BMI, kg/m^2^**	**Post-RT IL-18, pg/mL**						
<30	NA	Referent		Referent		Referent	
≥30	NA	**2.04 (1.14, 3.64)**	**0.016**	**2.20 (1.34, 3.61)**	**0.002**	1.39 (0.80, 2.39)	0.242
NA	<5.26	Referent		Referent		Referent	
NA	≥5.26	1.36 (0.76, 2.43)	0.295	1.51 0.93, 2.45)	0.095	1.61 (0.964 2.76)	0.086
<30	<5.26	Referent		Referent		Referent	
<30	≥5.26	1.37 (0.60, 3.09)	0.455	1.40 (0.73, 2.67)	0.309	1.46 (0.72, 2.94)	0.297
≥30	<5.26	2.15 (0.91, 5.09)	0.083	**2.** **10 (** **1.02, 4.** **32)**	**0.045**	1.20 (0.52, 2.75)	0.670
≥30	≥5.26	**2.56 (1.15, 5.69)**	**0.021**	**3.01 (1.54, 5.91)**	**<0.001**	**2.09 (1.00, 4.35)**	**0.048**
**BMI, kg/m^2^**	**Change of IL-18 by RT**						
<30	NA	Referent		Referent		Referent	
≥30	NA	**2.04 (1.14, 3.64)**	**0.016**	**2.20 (1.34, 3.61)**	**0.002**	1.39 (0.80, 2.39)	0.242
NA	<0.05	Referent		Referent		Referent	
NA	≥0.05	0.97 (0.54, 1.72)	0.904	0.89 (0.55, 1.44)	0.629	1.10 (0.64, 1.89)	0.722
<30	<0.05	Referent		Referent		Referent	
<30	≥0.05	0.78 (0.35, 1.83)	0.612	0.77 (0.40, 1.48)	0.428	1.02 (0.50–2.07)	0.967
≥30	<0.05	1.62 (0.70, 3.75)	0.265	1.81 (0.90, 3.66)	0.097	1.24 (0.56, 2.75)	0.603
≥30	≥0.05	1.96 (0.88, 4.37)	0.101	**2.02 (1.00, 4.07)**	**0.049**	1.56 (0.72, 3.38)	0.261

Note. Pre-RT pain was defined as a pre-RT pain score ≥ 4, post-RT pain as a post-RT pain score ≥ 4, and RT-related pain as an increase from <4 pre-IIRT to ≥4 post-RT. Odds ratios (ORs) and 95% confidence intervals (CIs) were obtained from multivariable logistic regression models adjusted for race/ethnicity, age group, RT fractionation, and clinical tumor stage. IL-18 values were log-transformed and dichotomized at the median (pg/mL). Boldface indicates *p* < 0.05.

## Data Availability

The original contributions presented in this study are included in the article. Further inquiries can be directed to the corresponding author.

## Supplementary Materials

The following supporting information can be downloaded at: https://www.mdpi.com/article/10.3390/cancers18071100/s1, Figure S1: Proposed IL-18–Inflammasome–Pain Signaling Pathway; Figure S2. ROC Curves for Prediction of Pre-RT Pain Using Pre-RT IL-18 Models. Figure S3. ROC Curves for Prediction of Post-RT Pain Using Pre-RT IL-18 Models. Figure S4. ROC Curves for Prediction of RT-Related Pain—Using Pre-RT IL-18 Models.

## Abbreviations

The following abbreviations are used in this manuscript:

AA	African American
AJCC	American Joint Committee on Cancer
ANOVA	Analysis of Variance
BMI	Body Mass Index
BPI	Brief Pain Inventory
CI	Confidence Interval
CV	Coefficient of Variation
ER	Estrogen Receptor
HER2	Human Epidermal Growth Factor Receptor 2
HW	Hispanic White
IL-18	Interleukin-18
NLRP3	NOD-Like Receptor Family Pyrin Domain Containing 3
NHW	Non-Hispanic White
NSABP	National Surgical Adjuvant Breast and Bowel Project
OR	Odds Ratio
PR	Progesterone Receptor
QOL	Quality of Life
RT	Radiotherapy
RTOG	Radiation Therapy Oncology Group
SD	Standard Deviation
TNF	Tumor Necrosis Factor
