# Supplementary material for: Interleukin-18 as a Potential Biomarker for Radiotherapy-Related Pain in Breast Cancer: Implications for Personalized Pain Management
Source: Cancers (Basel). 2026 Mar 28;18(7):1100. doi: 10.3390/cancers18071100 (PMC13072096; doi:10.3390/cancers18071100)
Supplement: Supplementary file 1 [file cancers-18-01100-s001.zip › cancers-4176162-supplementary.pdf]

Supplementary Figure S1. Proposed IL-18–Inflammasome–Pain Signaling Pathway

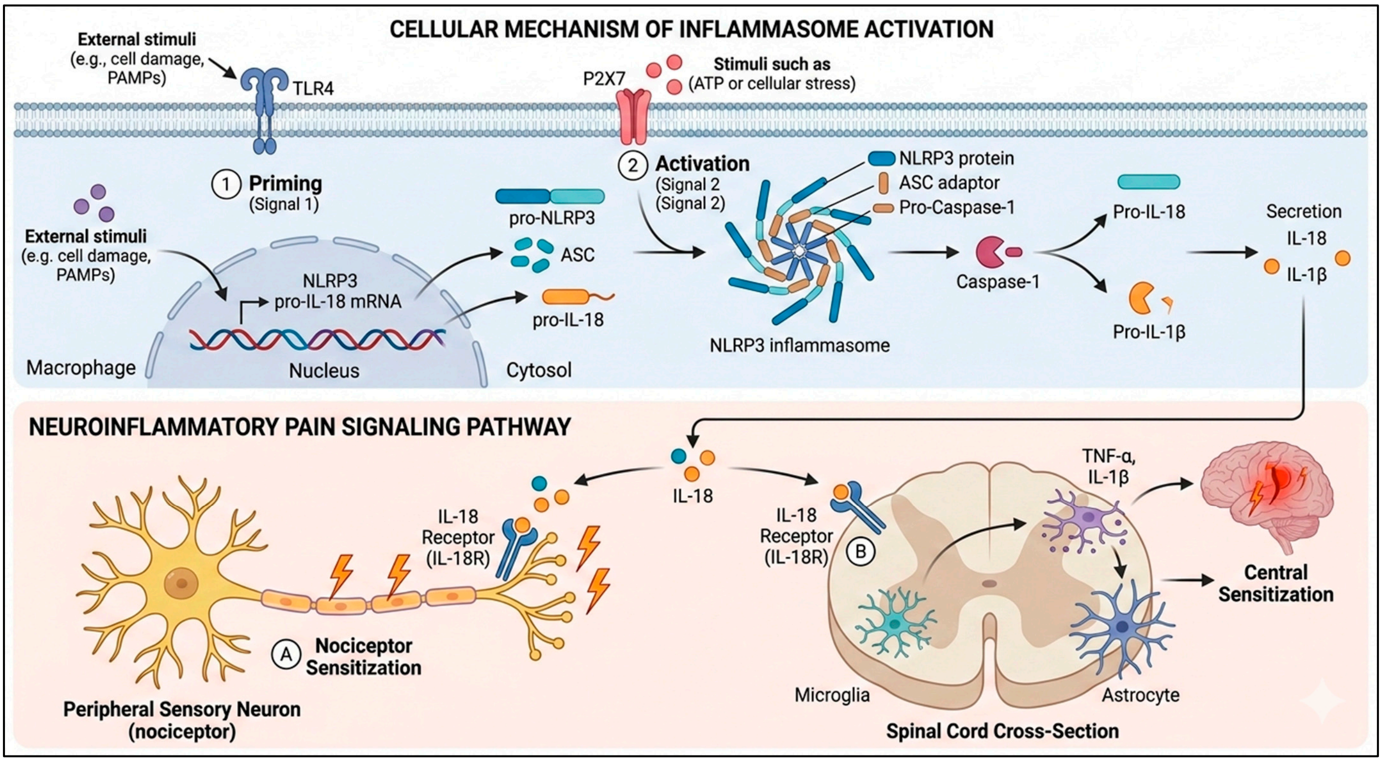

NOTE. RT-induced cellular damage and oxidative stress may activate NLRP3 inflammasome signaling pathways, leading to caspase-1 activation and release of pro-inflammatory cytokine IL-18. Elevated IL-18 promotes inflammatory signaling and nociceptive sensitization, potentially contributing to RT-related pain. *Figure generated using GEMINI.*

Supplementary Figure S2. ROC Curves for Prediction of Pre-RT Pain Using Pre-RT IL-18 Models

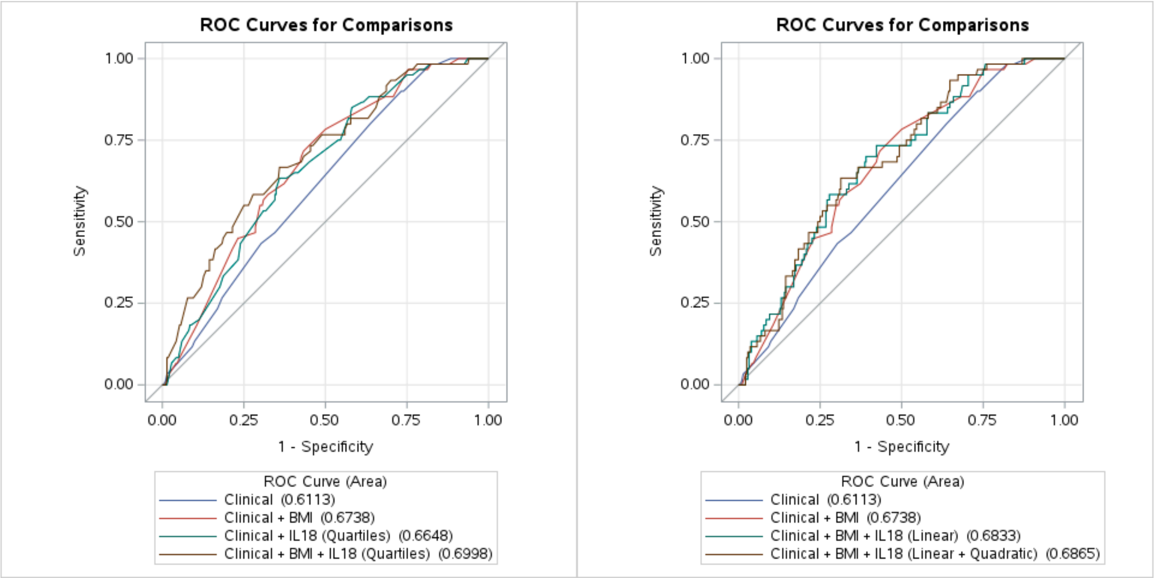

NOTE. Clinical model included age category, race/ethnicity, radiotherapy (RT) fractionation, and clinical tumor stage. Pre-RT pain was defined as a pain score ≥ 4 before RT. AUCs (95% CI) were 0.611 (0.541–0.682) for the clinical model, 0.674 (0.606–0.742) for clinical + BMI, 0.665 (0.596–0.734) for clinical + IL-18 quartiles, and 0.700 (0.629–0.771) for clinical + BMI + IL-18 quartiles. For continuous models, AUCs were 0.683 (0.615–0.752) for clinical + BMI + IL-18 (linear) and 0.687 (0.619–0.754) for clinical + BMI + IL-18 (linear + quadratic).

Supplementary Figure S3. ROC Curves for Prediction of Post-RT Pain Using Pre-RT IL-18 Models

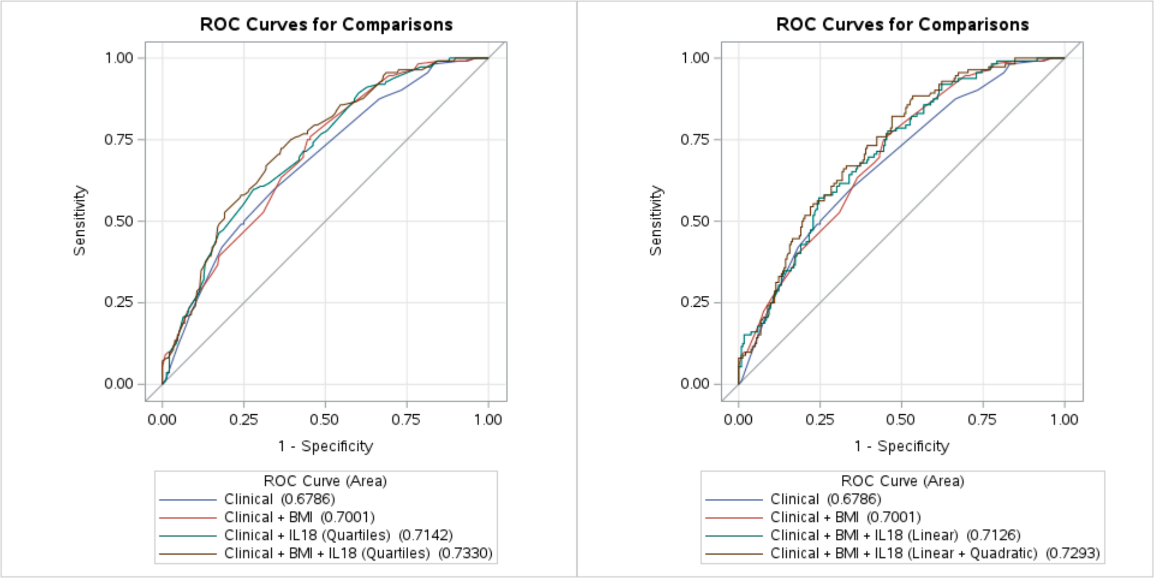

NOTE. Clinical model included age category, race/ethnicity, RT fractionation, and clinical tumor stage. Post-RT pain was defined as a pain score ⩾ 4 after RT. AUCs (95% CI) were 0.679 (0.621 – 0.736) for the clinical model, 0.700 (0.644 – 0.756) for clinical + BMI, 0.714 (0.659 – 0.770) for clinical + IL-18 quartiles, and 0.733 (0.679 – 0.787) for clinical + BMI + IL-18 quartiles. For continuous models, AUCs were 0.713 (0.657–0.768) for clinical + BMI + IL-18 (linear) and 0.729 (0.676–0.783) for clinical + BMI + IL-18 (linear + quadratic).

Supplementary Figure S4. ROC Curves for Prediction of RT-Related Pain Using Pre-RT IL-18 Models

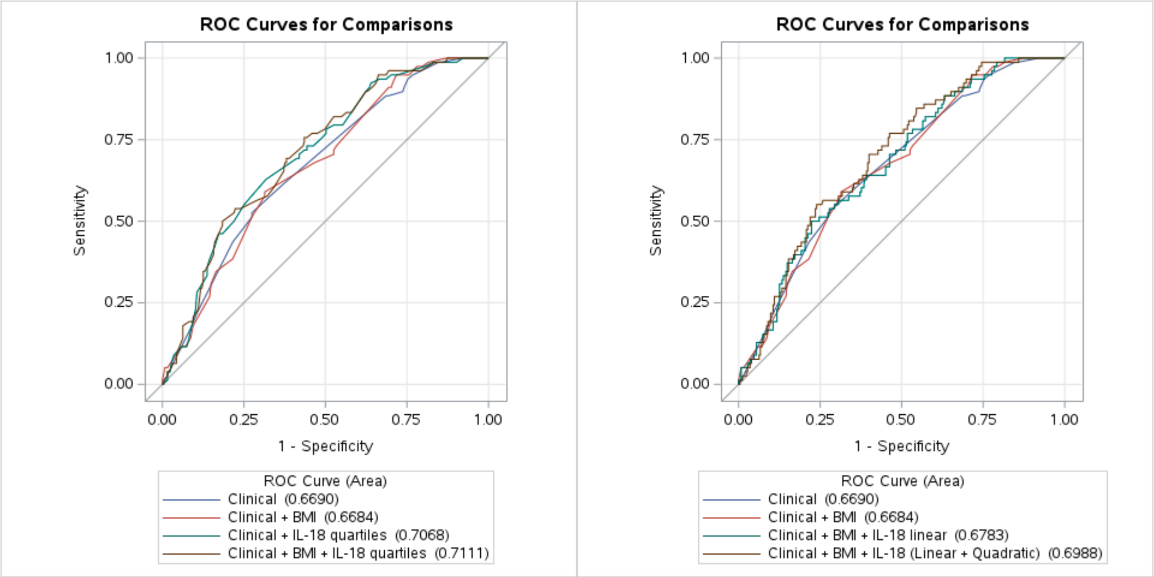

NOTE. Clinical model included age category, race/ethnicity, RT fractionation, and clinical tumor stage. RT-related pain was defined as an increase from < 4 pre-RT to ≥ 4 post-RT. AUCs (95% CI) were 0.669 (0.605–0.733) for the clinical model, 0.668 (0.604–0.732) for clinical + BMI, 0.707 (0.645–0.769) for clinical + IL-18 quartiles, and 0.711 (0.650–0.772) for clinical + BMI + IL-18 quartiles. For continuous models, AUCs of 0.678 (0.614–0.742) for clinical + BMI + IL-18 (linear) and 0.699 (0.637–0.760) for clinical + BMI + IL-18 (linear + quadratic).
